# Event-Based Visual/Inertial Odometry for UAV Indoor Navigation

**DOI:** 10.3390/s25010061

**Published:** 2024-12-25

**Authors:** Ahmed Elamin, Ahmed El-Rabbany, Sunil Jacob

**Affiliations:** 1 Civil Engineering Department, Faculty of Engineering and Architectural Science, Toronto Metropolitan University, Toronto, ON M5B 2K3, Canada; rabbany@torontomu.ca; 2 Civil Engineering Department, Faculty of Engineering, Zagazig University, Zagazig 10162, Egypt; 3 SOTI Aerospace, SOTI Inc., Mississauga, ON L5N 8L9, Canada; sunil.jacob@soti.net

**Keywords:** UAV, event camera, visual–inertial odometry, navigation

## Abstract

Indoor navigation is becoming increasingly essential for multiple applications. It is complex and challenging due to dynamic scenes, limited space, and, more importantly, the unavailability of global navigation satellite system (GNSS) signals. Recently, new sensors have emerged, namely event cameras, which show great potential for indoor navigation due to their high dynamic range and low latency. In this study, an event-based visual–inertial odometry approach is proposed, emphasizing adaptive event accumulation and selective keyframe updates to reduce computational overhead. The proposed approach fuses events, standard frames, and inertial measurements for precise indoor navigation. Features are detected and tracked on the standard images. The events are accumulated into frames and used to track the features between the standard frames. Subsequently, the IMU measurements and the feature tracks are fused to continuously estimate the sensor states. The proposed approach is evaluated using both simulated and real-world datasets. Compared with the state-of-the-art U-SLAM algorithm, our approach achieves a substantial reduction in the mean positional error and RMSE in simulated environments, showing up to 50% and 47% reductions along the *x*- and *y*-axes, respectively. The approach achieves 5–10 ms latency per event batch and 10–20 ms for frame updates, demonstrating real-time performance on resource-constrained platforms. These results underscore the potential of our approach as a robust solution for real-world UAV indoor navigation scenarios.

## 1. Introduction

Indoor navigation has recently received growing research interest due to its potential use in critical applications, such as medical care, search and rescue operations, and industrial management and inspections. In global navigation satellite system (GNSS)-denied environments, such as indoor environments, navigation is complex and challenging. Various sensors have been adopted for indoor navigation, such as inertial measurement units (IMUs), cameras, light detection and ranging (LiDAR), and ultra-wideband (UWB). The latter sensor is used to localize a system based on signal parameters received from fixed nodes of known locations. The most common signal parameters are the time of arrival, angle of arrival, and received-signal strength indication [1]. In [2,3,4], for example, a UWB-based system was utilized to navigate a UAV in indoor environments. However, UWB-based systems require additional infrastructure, which may not be cost-effective. LiDAR navigation, on the other hand, has traditionally been performed using either a 2D or 3D LiDAR sensor. Bachrach et al. [5] developed an unmanned aerial system (UAS) equipped with a 2D LiDAR sensor for navigation in unstructured indoor environments. Although 2D LiDAR navigation can operate in real-time, it cannot acquire height information and lacks the ability to construct a 3D map. On the other hand, in 3D LiDAR navigation, a complete real-time map can be constructed. An example of a commonly used 3D LiDAR navigation algorithm is LiDAR odometry and mapping (LOAM), which is ranked third on the KITTI data benchmark [6]. Light-LOAM introduced a computationally efficient LiDAR-based SLAM method designed for real-time performance on resource-constrained platforms [7]. In [8,9], a LiDAR/IMU fusion algorithm was proposed to localize a UAV in indoor environments. Despite LiDAR’s high accuracy and update frequency, the main challenges of using LiDAR sensors are the high price, large size, and power consumption [10].

Three types of cameras are widely used in visual sensor-based navigation: monocular, stereo and RGB-D. Monocular camera navigation is divided into three categories: the direct method, the feature point-matching method, and the semi-direct method [11]. Large-scale direct monocular simultaneous localization and mapping (LSD-SLAM), which estimates the camera motion using the gradient of the pixels, is one of the well-known direct methods [12]. Consequently, detecting feature points is unnecessary. Also, it can construct a semi-dense map. A typical representative of the point-matching method is the oriented fast and rotated brief SLAM (ORB-SLAM) [13], which was developed and formed on parallel tracking and mapping [14]. ORB-SLAM3 extends the original ORB-SLAM framework to support visual, visual–inertial, and multi-map SLAM for monocular, stereo, and RGB-D camera configurations, enhancing robustness and accuracy across diverse environments [15]. Semi-direct visual odometry (SVO) is an example of the semi-direct method [16], which combines direct and feature-point methods. Stereo camera systems are usually equipped with multiple lenses. Multiple lenses simultaneously capture the scene from multiple directions and then perform feature matching to restore more accurate 3D information about the environment [17]. The RGB-D cameras are equipped with a camera and an infrared transmitter and receiver, which can obtain the scene’s depth information [18]. At present, some mature RGB-D camera navigation solutions are available, such as RTAB-MAP [19] and dense visual odometry SLAM (DVO-SLAM) [20]. However, all these traditional cameras suffer from illumination changes in sensitivity, motion blur, and limited dynamic range.

A novel type of camera sensor called an event camera has recently attracted much attention from academia and industry [21]. It offers great advantages over conventional cameras, including high temporal resolution (in the range of μs), low latency, low power consumption, and a high dynamic range (140 dB vs. 60 dB) [21]. Event cameras are asynchronous sensors that represent a breakthrough in how visual information is acquired because they sample light based on the scene changes rather than at a rate an external clock specifies without any relation to the captured scene. Event cameras respond to brightness changes in the viewed scene asynchronously and independently for every pixel. Therefore, an event camera’s output is a variable data rate sequence of digital events. Each event represents a change in brightness at a specific pixel at a particular time.

In [22,23,24,25], it was shown that pose estimation is possible using only an event camera. For example, Kim et al. [22] proposed real-time 3D reconstruction and tracking, while Rebecq et al. [23] introduced a geometric approach for 6-DOF tracking. In [24], a method for range–visual–inertial odometry was presented, and in [25], an adaptive filtering approach was proposed to enhance the robustness and accuracy in dynamic environments. Events and inertial measurements were fused using a continuous-time framework [26]. However, this approach is not suitable for real-time applications due to the computational cost introduced by continuous optimization. Unlike traditional keyframe-based methods, this approach requires continuous spline fitting and optimization over an extended time window, leading to increased processing time and memory usage. This makes it unsuitable for real-time UAV navigation. In [27], an event-based visual–inertial odometry (EVIO) approach was proposed. An iterative expectation-maximization technique was used to track features in the event stream, and the feature tracks were then fed to an EKF filter to estimate the camera pose. EVIO can operate in real-time but for limited-speed motion. In [28], a real-time event-based VIO pipeline was proposed. This approach used keyframe-based nonlinear optimization to fuse the tracked features with the IMU measurements. They estimated the accumulated drift as the distance between the first and last positions, which reached 50 cm. In [29], event-based stereo visual odometry (ESVO) was developed, which addressed event-based stereo VO by maximizing spatio-temporal consistency across stereo event streams, allowing real-time 3D mapping and pose estimation even in high dynamic range and high-speed scenarios. Recent advances, such as ESVIO [30], leveraged event-based stereo VIO by integrating events’ depth, time–surface images, and IMU measurements in a sliding window framework, achieving high accuracy and robustness for state estimation in dynamic environments.

Feature detection and tracking are key steps in visual sensor-based navigation. Some commonly used image-based keypoint detectors, such as FAST [31] and the Harris corner detector [32], have been proposed for event cameras [33,34]. Also, histogram-of-oriented-gradients (HOG) detectors [35] have been developed for event camera recognition applications [36]. Generally, event-based trackers utilize binary feature templates, either predefined [37] or built from a set of events [38], to which they align events using iterative methods, such as the iterative closest point (ICP) [39]. However, those tracking approaches depend on motion direction and suffer from drift because of the event appearance variation over time [40]. Therefore, some approaches leveraged the advantages of frame-based and event-based combined cameras, such as DAVIS [41]. In [40,42,43], the algorithms automatically detect features on the frames and then track them asynchronously using events. Vidal et al. [44] proposed a state estimation pipeline that fuses data from an event camera, a standard camera, and an IMU. In their approach, the accumulated event frames are synced with the standard frames, where at each new standard frame a spatiotemporal window of events is selected. Subsequently, the features are extracted and tracked on both standard frames and event frames independently. Finally, the two sets of independent feature tracks are fused with IMU measurements through the back-end optimizer. They tested the system onboard a small UAV in an indoor environment under challenging conditions. The standard camera suffered severe motion blur at a relatively high speed, and the event camera showed reliable feature tracking. Therefore, the fusion of data from multiple sensors is essential for reliable and continuous localization for UAV indoor applications.

In this paper, an event-based visual–inertial odometry algorithm for event cameras is proposed. The proposed approach fuses data from an event camera, a standard camera, and an IMU for precise indoor navigation. Instead of tracking the features independently as in [38], features are detected on the standard frame images. Subsequently, to improve the tracking accuracy, the features are tracked on intermediate frames accumulated from events till the next standard frame. This ensures continuous tracking of the features in between standard frames, which enhances the VIO robustness. Finally, the feature tracks are fused with IMU measurements to estimate the sensor pose using nonlinear optimization. This paper is organized as follows: Section 2 presents the proposed event-based VIO approach. The experiments are explained in Section 3. Finally, conclusions are drawn in Section 4.

## 2. Event-Based VIO Approach

**Figure 1:**
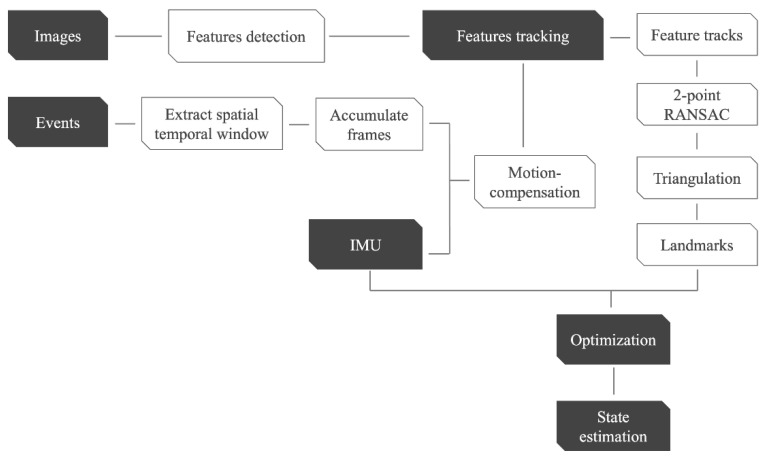
Workflow of the proposed event-based VIO.

The overall methodology flowchart is shown in Figure 1, which summarizes the steps of the proposed approach. The proposed VIO approach is inspired by [28,44]. The proposed approach consists of two main threads, the front-end thread and the back-end thread. In the front-end thread, features are detected and tracked on the standard images. Between the standard frames, the events are clustered in spatiotemporal windows and accumulated into frames. These intermediate accumulated frames are used to track the features between the standard frames. To ensure robust tracking, 2-point RANSAC is applied to filter out outliers based on the relative orientation between the last keyframe and the current frame. Subsequently, the filtered feature tracks are triangulated to their 3D locations. The back-end thread fuses the IMU measurements and the feature tracks to estimate the sensor states continuously. This approach is implemented based on the open source library dv-mono-vio-sample, by iniVation [45].

In contrast to traditional SLAM pipelines that rely on continuous keyframe-based nonlinear optimization, the proposed approach focuses on lightweight optimization, processing events asynchronously to maintain smooth pose estimation between frame intervals. This design reduces computational overhead, making it ideal for low-power and embedded systems, such as UAVs operating in indoor environments. Features are detected on standard frames and tracked continuously on intermediate event-accumulated frames without requiring re-detection at every time step. By leveraging spatiotemporal windows of events and aligning them using IMU-based motion compensation, ensuring accurate tracking while minimizing drift. The fusion of these multi-sensor inputs enhances localization accuracy without the need for intensive optimization, providing a scalable solution for real-time navigation.

For every standard frame at time $t_{k}$, the set of acquired events is clustered into overlapping spatiotemporal windows. The kth window, $W_{k}$, which comprises a set of events, can be described as follows:

(1) $$W_{k}=\left\{e_{kS},\ldots ,e_{kS+N-1}\right\},$$

where N is the window-size parameter and S is the step-size parameter, which determines the overlap between consecutive windows. Each spatiotemporal window’s duration is inversely related to the event rate. Each window, $W_{k}$, is accumulated on an event frame $I_{k}$ by projecting each event onto the image plane as follows:

(2) $$I_{k}\left(x\right)=\sum_{e_{j}\in W_{k}}\;\delta \left(x-x_{j}^{\prime }\right),$$

where I is the intensity at pixel x, $\delta \left(x\right)$ is the Kronecker delta, and $x_{j}^{\prime }$ is the event position after motion correction. Motion correction is important for reliable feature detection or tracking. Each event motion is corrected based on its timestamp by projecting the event, $e_{j}$, to the reference camera frame as follows:

(3) $$x_{i}^{\prime }=\pi_{0}\left(T_{t_{k},t_{i}}\left(Z\left(x_{i}\right)\pi_{0}^{-1}\left(x_{i}\right)\right)\right),$$

where $x_{i}$ is the pixel position of an event $e_{i}$; $\pi_{0}\left(.\right)\;$ is the projection model of the event camera acquired from intrinsic calibration; $T_{t_{k},t_{i}}\;$ is the transformation between the camera poses at times $t_{k}$ and $t_{i}$, estimated using the IMU measurements; and $Z\left(x_{i}\right)\;$ is the scene depth at pixel x_i_ and time t_i_, which can be estimated using the current landmarks’ median depth.

The FAST corner detector [31] was used for feature detection along with a bucketing grid to ensure an even distribution of the features over the image. Whenever a new standard frame is captured or the feature tracks drop below a specific threshold, new features are detected. The FAST threshold was set to 10, which balances sensitivity to corner detection and robustness to noise. Lower thresholds increase the sensitivity but may detect more false corners, while higher thresholds miss important features. Empirical testing on both simulated and real-world datasets showed that a threshold of 10 provides an optimal trade-off between detection accuracy and computational efficiency. Then, the detected features are tracked across standard frames and event frames using Lukas–Kanade tracking (LKT) [46]. Thereafter, two-point RANSAC [47] is used to filter out the feature tracks’ outliers based on the relative orientation between the last keyframe and the current frame.

In the back-end thread, feature tracks from standard frames and the event stream are fused with the IMU measurements to estimate the sensor state over time. The back-end implementation is based on OKVIS [48]. In contrast to EKF-based filters, OKVIS is a full smoothing technique based on nonlinear optimization on selected keyframes. This approach outperformed state-of-the-art techniques in terms of accuracy [49]. For more details about the back-end pipeline, the reader is referred to [48].

The visual–inertial odometry problem is a joint optimization of a cost function, which can be formulated as

(4) $$J=\sum_{i=0}^{1}\;\sum_{k=1}^{K}\;\sum_{j\in J(i,k)}\;e^{i,j,kT}W_{r}^{i,j,k}e^{i,j,k}+\sum_{k=1}^{K-1}\;e_{s}^{k^{T}}W_{s}^{k}e_{s}^{k}$$

This optimization function includes two weighted reprojection errors corresponding to the standard frames and the accumulated frames from the events in addition to inertial error terms, where i is the sensor index, k is the frame index, j is the landmark index, J(i,k) is the set of landmarks maintained in frame k by sensor i, $e^{i,j,k}$ is the reprojection error, $W_{r}^{i,j,k}$ is the information matrix of the landmarks, $e_{s}^{k}$ is the IMU error, and $W_{s}^{k}$ is the information matrix of the IMU error. The information matrix can be described as follows:

(5) $$e_{r}^{i,j,k}=z^{i,j,k}-\pi_{i}\left(T_{C_{i}S}^{k}T_{SW}^{k}l^{i,j}\right),$$

where $z^{i,j,k}$ is the image coordinates of landmark j on sensor i at frame k. The optimization is carried out on the standard frames as keyframes. Between frames, the IMU measurements along with the event frames that fall between frames are used to propagate the sensor state. Google Ceres optimizer [50] was applied to perform the optimization.

The current state is predicted based on the preceding state using the standard IMU kinematics and biases model presented in [51]. Then, the IMU error terms are calculated as the difference between the actual state and the predicted state. For orientation, a simple multiplicative minimal error is used.

## 3. Experimental Section

**Figure 2:**
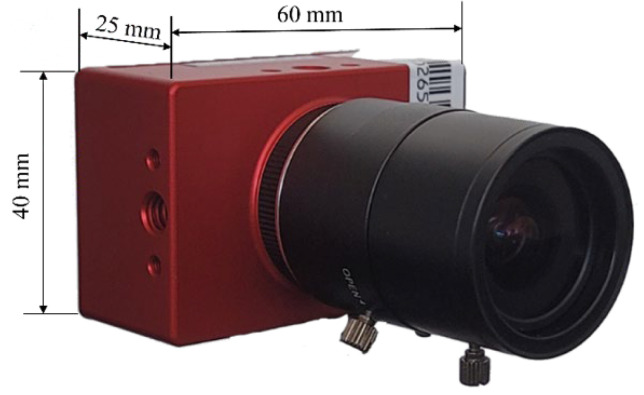
DAVIS346 event camera.

In this study, the DAVIS346 event camera [52] was used for data acquisition. The DAVIS346 integrates three sensors, namely an event camera, a standard RGB camera, and a six-axis IMU with a 1 kHz sampling rate. The event camera’s dynamic range is 120 db and a temporal resolution of 1 µs. On the other hand, the standard camera has a 55 db dynamic range. Both cameras have a 346 × 260 pixel image size, with a 113° horizontal field of view (FOV) and 97.7° vertical FOV. The DAVIS346 camera weighs 100 g without the lens, and its power consumption is less than 180 mA at 5V DC. Figure 2 shows an image of the DAVIS346 event camera.

**Figure 3:**
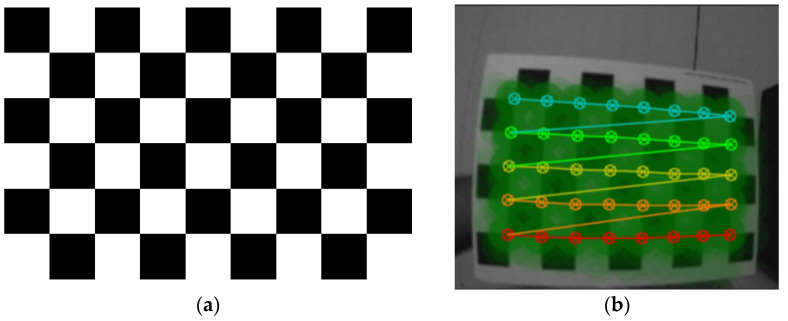
Study area camera calibration: (**a**) a 6 × 9 chessboard with a square size of 30 mm; and (**b**) an example of the detected pattern image.

The standard frame data were acquired at a 25 Hz frame rate, and the IMU data were acquired at a 200 Hz frame rate. Both cameras were calibrated prior to the data processing to determine the camera’s intrinsic parameters. The camera calibration was implemented using the DV software 1.3.1 [53]. A 30 mm^2^ 6 × 9 chessboard was used in the calibration, as shown in Figure 3a. By moving the chessboard in front of the camera, the calibration module in the DV software detects the chessboard pattern. Figure 3b shows an example of a detected pattern image. After capturing enough detected pattern images, the software calculates the calibration parameters and the maximum reprojection error, which was approximately 0.08 pixels.

### 3.1. Simulated Dataset

**Figure 4:**
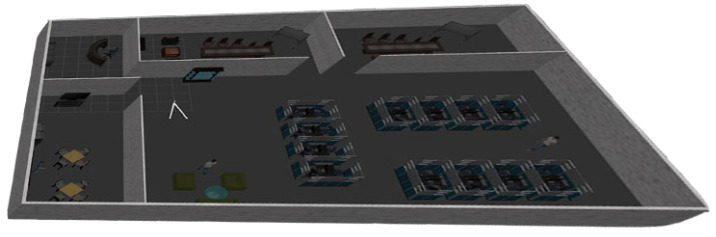
An office environment simulation layout.

To illustrate the effectiveness of the proposed approach, it was first evaluated on simulated data against the state-of-the-art algorithm ultimate SLAM (U-SLAM) [44]. An office environment was simulated using the Robot Operating System (ROS) and Gazebo, which spans an area of 600 m^2^, as shown in Figure 4. The simulation utilized the P230 UAV model, equipped with virtual sensors such as an RGB camera, an event camera, and an IMU. The DVS Gazebo plugin was employed to simulate the event camera data. Both the RGB and event cameras recorded images at a resolution of 240 × 180 pixels, with a horizontal FOV of 103° and a vertical FOV of 97.7°. The standard frame data were collected at 25 Hz, while the IMU data were recorded at a frequency of 50 Hz.

**Figure 5:**
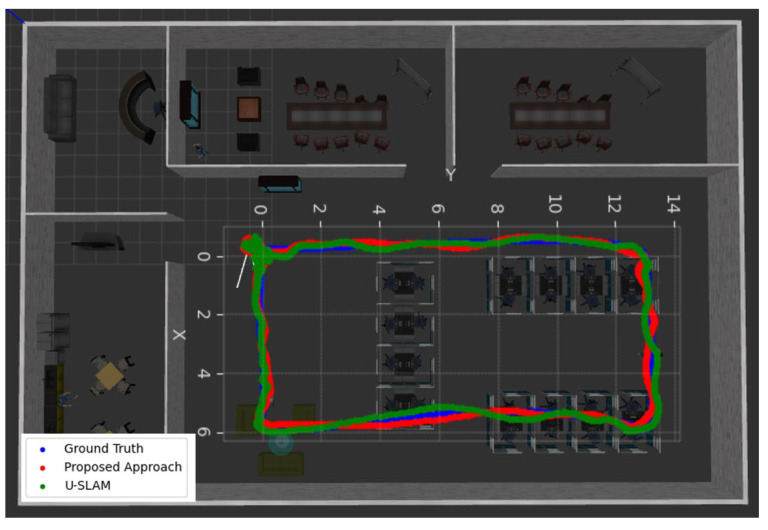
Simulated dataset: comparison of trajectories.

The proposed approach demonstrated significant performance improvements over U-SLAM in terms of position error metrics, as shown in Table 1 and Figure 5. It achieved smaller mean and root mean square error (RMSE) values across the *x*-, *y*- and *z*-axes compared to U-SLAM. Specifically, the mean positional error in the proposed approach was reduced by approximately 50% on the *x*-axis and 47% on the *y*-axis, while the RMSE exhibited comparable improvements, demonstrating a marked increase in overall accuracy. The maximum error recorded in the proposed method was also notably lower than U-SLAM, showcasing enhanced robustness and stability in simulated environments.

In addition to achieving significant reductions in the mean positional error and RMSE compared to U-SLAM, the proposed approach offers superior computational efficiency. By leveraging adaptive event-based tracking and selective keyframe updates, the system maintains continuous pose estimation with lower latency. Specifically, the proposed method achieved a processing latency of 5–10 ms per event batch and 10–20 ms for frame updates, compared to 15–30 ms per frame plus additional overhead for keyframe-based nonlinear optimization in U-SLAM. This reduction in computational demand makes the framework more suitable for resource-constrained devices, enabling deployment on platforms like lightweight UAVs. Furthermore, the efficient handling of events ensures robust performance in dynamic environments, providing consistent tracking between frames and mitigating the drift issues observed in systems that rely heavily on keyframes.

### 3.2. Ground-Based Dataset

In order to evaluate the proposed approach on real-world configurations, two datasets are presented: a ground dataset and a UAV-based dataset. Both datasets were acquired in an indoor environment following a predefined trajectory and ensuring a precise return to their initial starting positions. The ground-based dataset was captured through manual navigation with a DAVIS346 event camera. In the UAV-based dataset, a quadrotor platform equipped with the DAVIS346 camera was used for data acquisition.

**Figure 6:**
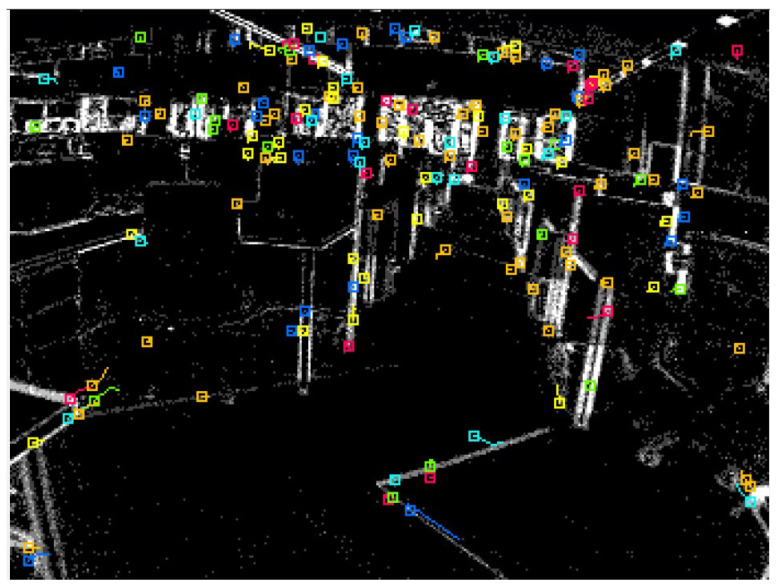
Ground-based dataset: an example of feature detection and tracking on an events accumulated frame.

**Figure 7:**
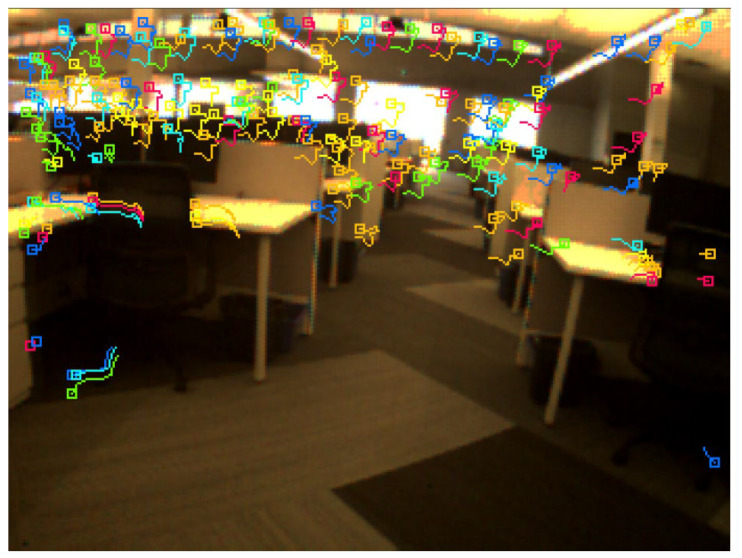
Ground-based dataset: an example of feature detection and tracking on a standard frame.

**Figure 8:**
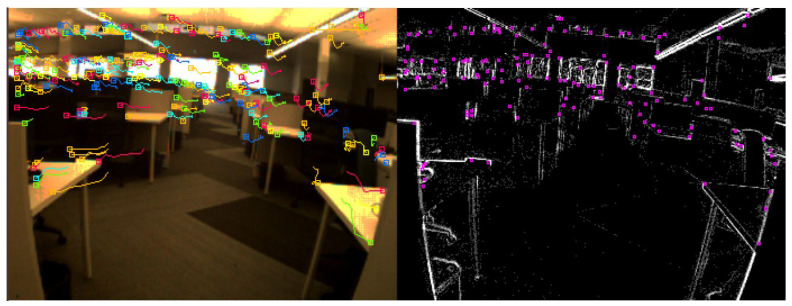
Ground-based dataset: an example of feature detection and tracking on combined events and standard frames.

The data were collected in an indoor environment with a complete absence of the GNSS signals. A predefined trajectory with precise coordinates was established and used as a reference. In addition, the trajectory was established to form a closed loop, which allows us to estimate the loop closer error and to use it as another evaluation metric. Three case studies were considered. The first of which was based on events only. In this case study, the tracking was achieved by accumulating frames from the event stream and then running the LKT frame-based tracker on them. Figure 6 shows an example of feature detection and tracking on an accumulated frame from the stream of events. The second case study, on the other hand, included the standard frames only. Figure 7 shows an example of feature detection and tracking on a standard frame. In both aforementioned cases, the feature tracks were fused with the IMU measurements using OKVIS in the back end. The third case study combined events, standard frames, and IMU measurements. In this case, the features were detected and tracked on the standard frame image, but to improve tracking quality, intermediate frames were accumulated from events and tracking was performed on those frames. Finally, the IMU measurements were fused with the feature tracks to estimate the camera states. Figure 8 shows an example of feature detection and tracking on the combined events and standard frames. The estimated trajectories from the three cases were compared to the ground truth.

**Figure 9:**
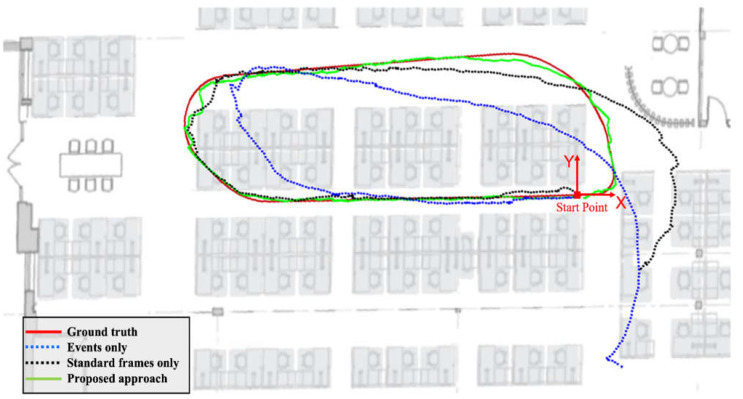
Ground-based dataset: comparison of trajectories.

Figure 9 compares the trajectories of the three case studies with respect to ground truth. Table 2 shows the error statistics of the position for the three navigation solutions. Table 3, on the other hand, shows the loop closing error for the three cases, which was calculated as the difference in coordinates between the first and last points. In Figure 9 and Table 2, it is noticeable that the event-only solution drifts significantly after a short time. The total RMSE of the event-only case was 2.33 m, while the loop closing error was 4.42 m. On the other hand, the standard frame-only solution sustained a relatively small error over a longer period, although it drifted by 2.45 m by the end of the trajectory. In addition, its overall RMSE was 1.45 m. Compared with the previous two case studies, the combined solution produced significantly fewer errors. The positioning RMSE decreased by 84% and 75% compared to the event-only and standard frame-only cases, respectively. The loop closing error of the combined case was 0.13 m for a 32 m trajectory, i.e., an accumulated drift of less than 1% compared to 13.8% and 8.0% in event-only and standard frame-only cases, respectively.

### 3.3. UAV-Based Dataset

**Figure 10:**
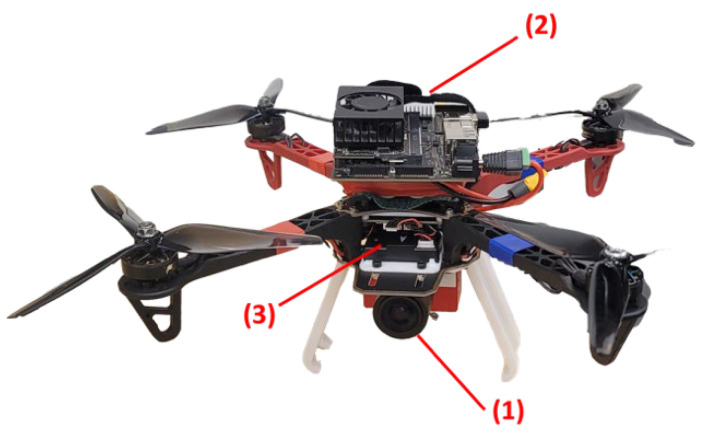
UAV used for the experiments. (1) DAVIS346 event camera. (2) NVIDIA Jetson Xavier computer. (3) Pixhawk 4 flight controller.

To assess the developed approach in a real-world scenario, it was tested using a dataset that was captured by a UAV. The UAV was assembled using the DJI F330 frame and was equipped with a DAVIS346 event camera, a Pixhawk 4 flight controller [54], and an NVIDIA Jetson Xavier computer. Figure 10 illustrates how these components are rigidly fixed to the UAV platform, with the event camera positioned at the front, facing forward.

**Figure 11:**
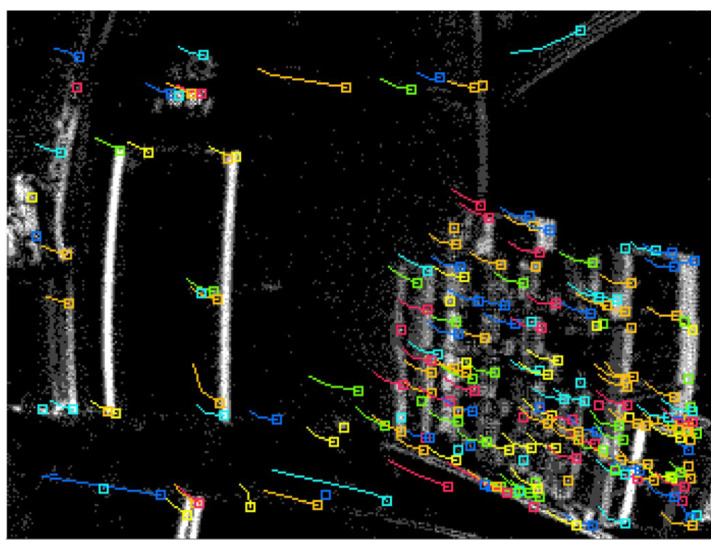
UAV-based dataset: an example of feature detection and tracking on an events accumulated frame.

**Figure 12:**
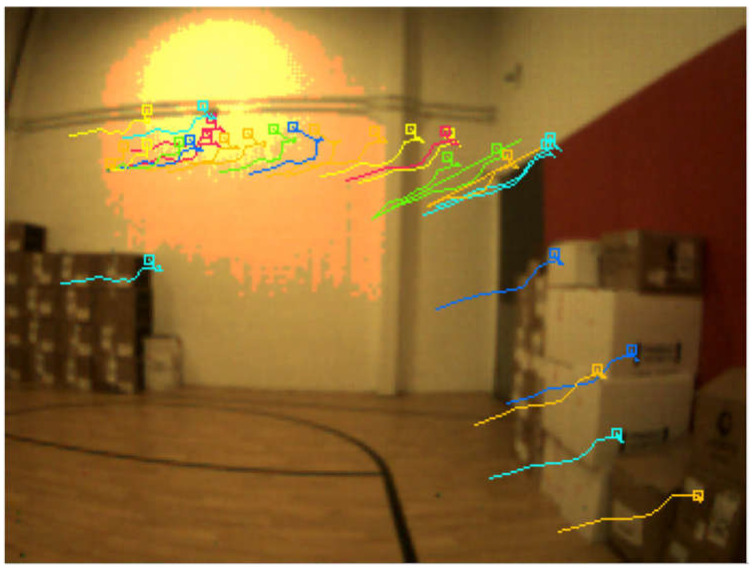
UAV-based dataset: an example of feature detection and tracking on a standard frame.

**Figure 13:**
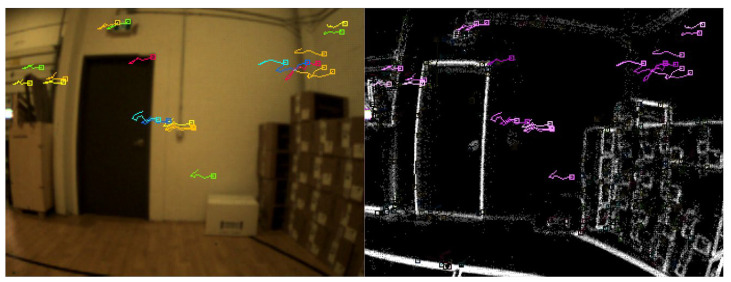
UAV-based dataset: an example of feature detection and tracking on combined events and standard frames.

**Figure 14:**
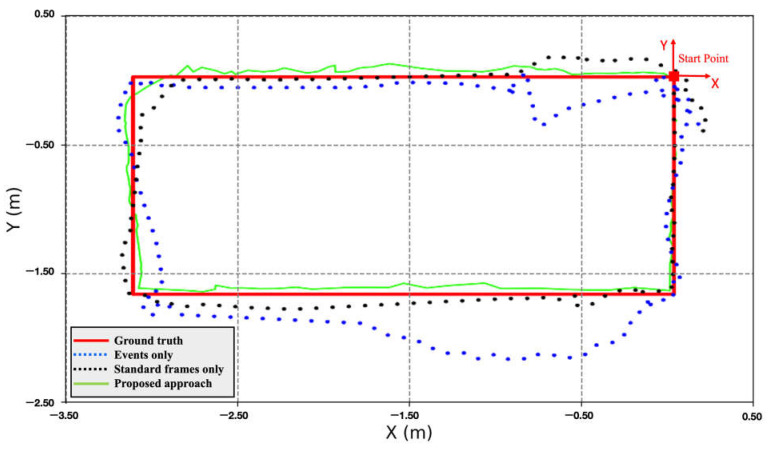
UAV-based dataset: comparison of trajectories.

Similar to the ground-based dataset, the UAV flew indoors with no GNSS signals available, following a predefined trajectory with a total length of 9 m. The same three case studies were considered, namely event-only case, frames-only case, and combined case. Figure 11, Figure 12 and Figure 13 visually depict feature detection and tracking in each case. The resulting trajectories were then compared to the ground truth, as illustrated in Figure 14. Table 4 provides error statistics for the position in the three navigation solutions, while Table 5 shows the loop closing error, calculated as the difference in coordinates between the first and last points.

In the event-only case, the solution exhibited some drift after a short duration. The total RMSE for the event-only case was 0.55 m, with a loop closing error of 0.57 m. Conversely, the standard frame-only solution sustained a relatively small error over a more extended period, drifting by 0.41 m by the end of the trajectory. Its overall RMSE was 0.33 m. Notably, the combined solution yielded significantly fewer errors compared to the individual cases. The positioning RMSE decreased by 89% and 82%, compared to the event-only and standard frame-only cases, respectively. The loop closing error of the combined case was 0.04 m for a 9 m trajectory, indicating an accumulated drift of less than 1% compared to 6.3% and 4.5% in event-only and standard frame-only cases, respectively.

## 4. Conclusions

In this study, an event-based visual–inertial odometry algorithm for event cameras was developed for indoor navigation. The proposed approach consisted of two main threads: the front-end and the back-end. The front-end utilized the FAST corner detector for feature detection and the Lukas–Kanade tracking for feature tracking. The back-end, inspired by OKVIS, integrated events, frames, and inertial measurements to estimate the sensor state over time. The performance of the proposed approach was evaluated using simulated and real-world datasets. On simulated data, our approach outperformed U-SLAM, reducing mean positional error and RMSE by up to 50% and 47% on the *x*- and *y*-axes, respectively, with smaller maximum error. It also demonstrated real-time feasibility with 5–10 ms latency per event batch and 10–20 ms for frame updates, making it scalable for resource-constrained platforms such as UAVs.

For real-world DAVIS346-based datasets, three case studies were considered: event-only, frame-only, and combined cases. Two scenarios were considered: a ground-based dataset captured via manual navigation and a UAV-based dataset recorded with a quadrotor platform. The event-only scenario showed drift after a short duration, while the frame-only solution performed better. The combined case achieved substantial improvements in pose estimation, with RMSE reductions of 84% and 75% in the ground-based dataset and 89% and 82% in the UAV-based dataset, relative to event-only and frame-only cases, respectively. The accumulated drift of the combined case was under 1%. These results confirm that the proposed approach provides efficient and accurate indoor navigation, balancing performance with computational demands, and supporting real-time deployment on embedded systems.

## Figures and Tables

**Table 1:** Simulated dataset: comparison of the three cases’ position (m) error statistics.

	U-SLAM			Proposed Approach		
	Mean	RMSE	Max	Mean	RMSE	Max
X	0.18	0.25	0.33	0.09	0.18	0.28
Y	0.19	0.24	0.42	0.1	0.19	0.38
Z	0.35	0.40	0.64	0.1	0.26	0.27
Total		0.31			0.21	

**Table 2:** Ground-based dataset: comparison of the three cases’ position (m) error statistics.

	Event-Only Case			Frame-Only Case			Combined Case		
	Mean	RMSE	Max	Mean	RMSE	Max	Mean	RMSE	Max
X	1.4	1.60	2.52	0.77	1.33	2.44	−0.17	0.35	0.92
Y	−0.71	1.70	4.12	−0.10	0.59	1.62	−0.05	0.13	0.27
Total		2.33			1.45			0.37	

**Table 3:** Ground-based dataset: comparison of the three cases closer error (m).

	Event-Only Case	Frame-Only Case	COMBINED CASE
X	1.58	1.95	0.12
Y	−4.13	−1.62	−0.06
Total	4.42	2.54	0.13

**Table 4:** UAV-based dataset: comparison of position (m) error statistics.

	Event-Only Case			Frame-Only Case			Combined Case		
	Mean	RMSE	Max	Mean	RMSE	Max	Mean	RMSE	Max
X	0.14	0.46	0.65	0.14	0.31	0.59	−0.01	0.04	0.14
Y	−0.1	0.30	0.33	0.01	0.11	0.24	0.04	0.05	0.09
Total		0.55			0.33			0.06	

**Table 5:** UAV-based dataset: comparison of closer error (m).

	Event-Only Case	Frame-Only Case	Combined Case
X	0.30	0.19	−0.04
Y	−0.48	−0.36	0.01
Total	0.57	0.41	0.04

## Data Availability

The data presented in this study are not publicly available.
